# Metabolomic Profiling in Children with Celiac Disease: Beyond the Gluten-Free Diet

**DOI:** 10.3390/nu15132871

**Published:** 2023-06-25

**Authors:** Rafael Martín-Masot, María Jiménez-Muñoz, Marta Herrador-López, Víctor Manuel Navas-López, Elia Obis, Mariona Jové, Reinald Pamplona, Teresa Nestares

**Affiliations:** 1 Pediatric Gastroenterology and Nutrition Unit, Hospital Regional Universitario de Malaga, 29010 Málaga, Spain; rafammgr@gmail.com (R.M.-M.); herradorlopezm@gmail.com (M.H.-L.); victor.navas@gmail.com (V.M.N.-L.); 2 Institute of Nutrition and Food Technology “José MataixVerdú” (INYTA), Biomedical Research Centre (CIBM), University of Granada, 18071 Granada, Spain; nestares@ugr.es; 3 Department of Experimental Medicine, Lleida Biomedical Research Institute (IRBLleida), University of Lleida (UdL), 25198 Lleida, Spain; eobis@irblleida.cat (E.O.); mariona.jove@udl.cat (M.J.); reinald.pamplona@udl.cat (R.P.); 4 Department of Physiology, Faculty of Pharmacy, University of Granada, 18071 Granada, Spain

**Keywords:** celiac disease, gluten-free diet, metabolomics, children, immune, intestinal

## Abstract

Celiac disease (CD) is included in the group of complex or multifactorial diseases, i.e., those caused by the interaction of genetic and environmental factors. Despite a growing understanding of the pathophysiological mechanisms of the disease, diagnosis is still often delayed and there are no effective biomarkers for early diagnosis. The only current treatment, a gluten-free diet (GFD), can alleviate symptoms and restore intestinal villi, but its cellular effects remain poorly understood. To gain a comprehensive understanding of CD’s progression, it is crucial to advance knowledge across various scientific disciplines and explore what transpires after disease onset. Metabolomics studies hold particular significance in unravelling the complexities of multifactorial and multisystemic disorders, where environmental factors play a significant role in disease manifestation and progression. By analyzing metabolites, we can gain insights into the reasons behind CD’s occurrence, as well as better comprehend the impact of treatment initiation on patients. In this review, we present a collection of articles that showcase the latest breakthroughs in the field of metabolomics in pediatric CD, with the aim of trying to identify CD biomarkers for both early diagnosis and treatment monitoring. These advancements shed light on the potential of metabolomic analysis in enhancing our understanding of the disease and improving diagnostic and therapeutic strategies. More studies need to be designed to cover metabolic profiles in subjects at risk of developing the disease, as well as those analyzing biomarkers for follow-up treatment with a GFD.

## 1. Introduction

Celiac disease (CD) is included in the group of complex or multifactorial diseases, i.e., those caused by the interaction of genetic and environmental factors [1]. It is a chronic disease whose severity and digestive and/or systemic symptoms show great variability among patients. However, the common characteristic to all of them is an exacerbated immune response to gluten and related proteins, so this systemic disorder is considered an immune-mediated disease. In fact, patients are characterized by the presence of high titers of specific antibodies and the vast majority are carriers of the DQ2 and/or DQ8 haplotypes of the major histocompatibility complex (Human Leukocyte Antigen, HLA class II), responsible for antigen presentation by the immune system [2,3,4]. In addition to gluten intake and the presence of risk alleles in HLA, the occurrence of intestinal and extra-intestinal symptoms in CD requires the activation of both types of immune response, innate and adaptive, and this overactivation of the immune system is observed at the intestinal level as well as at the peripheral and systemic levels [4].

The main gap in the knowledge of the pathogenesis of CD is an explanation of why 25–35% of the world’s healthy population has these haplotypes, but only about 1–3% will develop CD [5,6]. It is possible that other environmental and genetic factors influence an individual’s ability to induce and control the innate response and an individual’s susceptibility to gluten.

Even though CD is actually one of the most frequent genetic diseases, affecting 1–3% of the world’s population, and to a greater extent women and children [7], it is clearly underestimated and underdiagnosed. Despite advances in knowledge and the development of serological tests, CD continues to be difficult and costly to diagnose, largely due to the systemic nature of CD, the lack of specificity of its clinical manifestations and the existence of silent or latent forms [8,9]. The problem is that untreated celiacs or those whose diagnosis has been delayed, despite having no symptoms, may have an exacerbated and chronic activation of the immune system, which is uncontrolled for a longer time, leading to a worse prognosis, an accentuation of symptoms and the appearance of other autoimmune diseases such as type 1 diabetes or gluten-dependent hepatitis, which are frequent in CD patients [10,11]. Also, they may be affected by a variety of adverse consequences, some serious such as the development of malignant tumors [12]. Therefore, at the present time, the main challenge, like for other genetic-based diseases (such as some types of cancer and diabetes, among others), is to study in genetically predisposed individuals, on the one hand, which factors are involved in the development or not of CD and, on the other, to find biomarkers for its early diagnosis and follow-up. The idea is to avoid the side effects when the disease has already made its debut and even prevent its appearance, something relevant since it is currently incurable.

The only current treatment for CD is a lifelong strict gluten-free diet (GFD) that achieves a remission of symptoms within a few days or weeks and a restoration of intestinal villi and immune homeostasis within a few months. We ourselves have found that, after 18 months of a strict GFD follow-up, the celiac has most of the parameters equivalent to those of a healthy child [13,14,15]. This is an important finding, but, even so, the usual delay in diagnosis (because of its difficulty), in combination with the time it takes to stabilize the disease, is too long, and in this period they may develop complications that will result in sequelae that range from minor (e.g., permanent short stature, dental enamel failure and psychiatric problems) to serious, such as tumors, in the long term. The fact is that the GFD seems to play an important role in the pathogenesis of tumor development, since some studies have described a greater development of tumors the later the diagnosis is made and in patients who have not followed the GFD [16]. Thus, untreated CD is associated above all with T-cell lymphoma (EATL) [12] and small bowel adenocarcinoma [17], although a higher incidence of non-Hodgkin’s lymphoma and colorectal cancer has also been described [18,19].

As CD is a systemic and complex disease, it is necessary to look at it from different perspectives. Personalized or precision medicine refers to the application of biotechnology, genetic profiling, “omics” sciences and the incorporation of clinical and environmental factors to evaluate individual risks and design strategies for the prevention, diagnosis, treatment or follow-up of the disease at the right time and in the right patient, with the minimum toxicity and maximum possible efficacy. One of the sciences that has been booming in recent years is metabolomics, which deals with the study of chemical processes in which small molecules, called metabolites, which can be both endogenous and xenobiotic, are measured. These molecules give information about a metabolic process that has taken place in the organism, and metabolomics can be considered, from this perspective, as an approach to cellular metabolism that other “omics” sciences cannot provide [20,21]. In this sense, metabolomics is presented as a fast and non-invasive tool that could represent a step forward in the knowledge of many diseases through the study of the metabolic profile by obtaining what are known as “metabolomic fingerprints or signatures” resulting from the interaction of the genome, epigenome, transcriptome, proteome and the environment.

Metabolomics studies are especially relevant in those multifactorial and multisystemic pathological situations where the environmental factor plays a relevant role in the appearance and development of the disease. Technologies such as metabolomics could define the alterations that occur in the genetically predisposed individual, as well as after certain changes, such as the GFD, helping to better understand these complex interactions, and thus may be useful for the diagnosis and monitoring of CD [22]. In this review, we cover several articles highlighting the latest advancements in the field of metabolomics in pediatric CD. Specifically, we explore studies that examine plasma and urine samples, with a special focus on the role of the GFD.

The main objective of this work was to compile the results of recent studies on the metabolomic profile of pediatric celiac patients, both at diagnosis and throughout the establishment of the GFD and the evolution of the disease, as well as its comparison with healthy children with/without genetic risk of CD. The ultimate objective was to establish the foundations of current knowledge on the subject, enabling the development of future research, ideally with the creation of new biomarkers. These biomarkers would facilitate an optimized management of the disease beyond the GFD.

## 2. Materials and Methods

We obtained published studies related to the topic in MEDLINE or PubMed, Scopus, Embase and Web of Science. The narrative review was conducted in March and April 2023. Search terms used were “metabolomics”, “metabolome”, “celiac/coeliac disease”, “metabolites” “biomarkers”, “gluten free diet”. Filters applied were child: birth–18 years; infant: birth–23 months; infant: 1–23 months; newborn: birth–1 month; preschool child: 2–5 years; child: 6–12 years; adolescent: 13–18 years. Articles published in English or Spanish were selected for critical synthesis. We included studies carried out on blood (plasma/serum) and urine. The search was completed with a review of bibliographic references. Exclusion criteria included articles that lacked a comprehensive description of the study in their full texts, studies published in non-peer-reviewed journals, meta-analyses, reviews, protocols, editorials and letters to the editor and studies conducted on animal models.

## 3. Metabolomics Platforms

Clinical metabolomics is a rising field of clinical research that takes advantage of the great technological advances that have been developed in recent years. This relatively new discipline involves the systematic analysis of metabolites (small molecules involved in metabolic pathways) in biological samples such as blood, urine and tissues to identify and measure the unique metabolic profile of an individual [23,24,25]. This profile can provide valuable information about an individual’s health status, including disease diagnosis, prognosis and treatment response [26,27,28]. Clinical metabolomics integrates various analytical techniques, such as mass spectrometry (MS) and nuclear magnetic resonance (NMR) spectroscopy, to detect and quantify metabolites in biological samples [29,30,31]. The data obtained from metabolomic analysis can be used to develop personalized and precision medicine strategies, by identifying biomarkers and metabolic pathways associated with specific diseases or conditions [32].

Clinical metabolomics is of great importance in medical and biomedical research because it allows the identification of metabolic biomarkers that can be used for the early detection, diagnosis and monitoring of diseases [30]. By analyzing changes in metabolic profiles, clues can be obtained about the pathogenesis of diseases and the underlying molecular mechanisms [31,33,34,35,36,37,38,39]. Using this technique allows the scientific and clinical community to follow and evaluate a wide variety of physiological conditions, such as the physiological changes that occur during pregnancy or aging [40,41,42,43] as well as for the diagnosis and monitoring of different diseases such as diabetes, cancer or neurodegenerative diseases [44,45,46,47,48,49,50]. In addition, clinical metabolomics can also help to develop new drugs and improve the efficacy and safety of existing treatments by providing information on how patients metabolize and respond to drugs [51,52,53,54]. It has the potential to improve diagnostic accuracy, treatment efficacy and understanding of the biological mechanisms underlying diseases [55].

The two most common techniques used in data acquisition for metabolomics analyses are NMR and MS [55]. Table 1 shows some of the key differences between the two techniques [47,56,57,58,59,60]. The principle of NMR spectroscopy is based on the interaction between the magnetic moments of atomic nuclei and an external magnetic field [61,62]. When a sample is placed in a magnetic field and irradiated with radiofrequency energy, the nuclei absorb and re-emit energy at characteristic frequencies, which is used to obtain information about the chemical environment and structure of the molecules. In the case of clinical metabolomics, NMR spectroscopy is used to analyze biological samples containing a complex mixture of metabolites [29]. The sample is prepared by extracting the metabolites and dissolving them in a deuterated solvent to prevent interference from the solvent itself. The sample is then placed in an NMR spectrometer, where it is subjected to a strong magnetic field and radiofrequency energy [63].

MS is the other main technique used in clinical metabolomics to detect and quantify metabolites in biological samples such as blood, urine and tissues [64]. The principle of MS is based on the ionization of molecules and the separation of ions based on their mass-to-charge ratio (*m*/*z*) in a mass analyzer [65]. The sample is first ionized, usually by using an ionization source such as electrospray ionization (ESI), atmospheric pressure chemical ionization (APCI) or matrix-assisted laser desorption/ionization [66,67,68,69,70] (MALDI). The ions are then separated and detected by the mass analyzer, which produces a mass spectrum showing the abundance of ions at different *m*/*z* values. The sample is prepared by extracting the metabolites and separating them from other compounds that may interfere with the analysis. The extracted metabolites are then subjected to ionization and analyzed by MS. It can detect and quantify a wide range of metabolites with high sensitivity and specificity, making it a powerful tool for clinical metabolomics research [60]. In addition, MS can be coupled with chromatography techniques such as liquid chromatography (LC) or gas chromatography (GC), which allows for further separation and analysis of metabolites. This approach, known as LC-MS or GC-MS [71,72,73], is a common method used in clinical metabolomics to analyze complex samples [74]. The pitfalls of the technique are that sample processing is more complex and that costs per sample are higher than in NMR. However, MS is the technique that is used the most in clinical metabolomics research due to its ability to detect and quantify a wide range of metabolites with high sensitivity and specificity [58].

**Figure 1:**
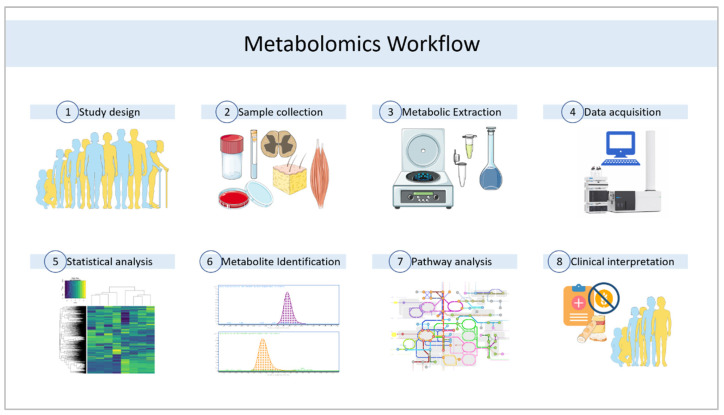
General workflow for clinical metabolomics studies. Author contributions. This figure was partly generated using Servier Medical Art, provided by Servier, licensed under a Creative Commons Attribution 3.0 unported license.

Both techniques, apart from using different detectors, also need appropriate sample processing according to the technique that will be used, as well as different data processing and analysis in each case [75]. Sample preparation is key in these analyses [60,76,77], as well as the selected approach for the experimental design, i.e., whether the metabolomic analysis is directed or not [29]. When we want to gain a global idea of the metabolic profile of a particular sample to open new questions or to see general changes, an untargeted analysis is used [43,78,79]. If we want to analyze only a more specific set of metabolites, which are usually related either by structure or by belonging to the same metabolic pathway, we use a targeted analysis [80,81]. In both cases, sample preparation and data analysis follow a slightly different workflow [82]. Figure 1 shows some of the key steps in clinical metabolomics approaches.

The clinical applications of metabolomics are many and diverse. Among them are the identification of biomarkers of diseases, the development of new therapies [28,83,84,85], the personalization of medical treatments [52,54], the early detection of diseases or the monitoring of the progression of diseases and response to treatment [86,87,88,89]. All of them have great value for the scientific and health community, as well as for all of society. In recent years, different technologies and methodologies for metabolomics have been developed and improved, which can help overcome some of the current challenges and enable significant advances in medical and biomedical research. Some of the new technologies and methodologies under development include ion mobility, metabolic flux analysis or separation-free MS techniques for direct infusion acquisition or metabolic imaging [90,91,92,93,94,95,96,97,98,99,100,101]. Although there are challenges in the implementation of clinical metabolomics, such as metabolite identification, the standardization of analysis techniques and the interpretation of complex data, new technologies and methodologies are constantly developing and can help overcome these barriers [29]. It is necessary to foster interdisciplinary collaboration to advance the field of clinical metabolomics, since the combination of knowledge and skills from different disciplines, such as biochemistry, bioinformatics, medicine and engineering, can lead to significant advances in the understanding of physiology and disease and in the development of personalized treatments.

## 4. Plasma Metabolomic Profile

### 4.1. CD’s Inherent Footprint and Role of the GFD

Several molecules have been proposed as potential CD biomarkers. In this regard, Auricchio et al., 2019 [102] found that the serum phospholipid profile is different in children who develop CD compared to healthy children with similar genetic profiles (specific celiac human HLA DQ2 or DQ8), even before the introduction of gluten to the diet at 4 months of age. They followed a cohort of children from families with a CD case from birth to 8 years of age, with sampling at 4 and 12 months of age (and at CD diagnosis in cases >24 months of age), finding in lipidomic analysis based on LC coupled with MS and multiple reaction monitoring (MRM) that the lipid profile is fairly constant in each individual, in both groups, suggesting that it could be constitutive. In the age-grouped analysis, they found that children who developed CD had increased lyso- and phosphatidylcholine (PC) serum levels (PC 40:4 showed the greatest difference between the two groups), as well as alkylacylphosphatidylcholine (PC-O). Specifically, two alkylacylphosphatidylcholipids (PC O-42:0 and PC O-38:3), together with breastfeeding and one phosphatidylcholine (PC 34:1), were defined as predictors of CD development. They found that phosphatidylethanolamines (PE) PE 34:1 and PE 36:1 were decreased in celiac patients.

The working group of Sen et al., 2019 [103] also applied lipidomics in the study of a cohort of Finnish children in the context of the Type 1 Diabetes Prediction and Prevention study. Based on MS and comparing plasma samples from children who developed CD with plasma samples from healthy controls, matched for HLA risk, sex and age, they found that CD progressors (children who developed CD) had increased triacylgycerols (TGs) of low carbon number, double-bond count plasma levels and decreased phosphatidylcholines and cholesterol esters levels at 3 months of age compared to controls. These differences were not apparent at birth (cord blood) and exacerbated with age. It is proposed that the increase in TGs of low carbon number and double-bond count is due to de novo lipogenesis compensating for lipid malabsorption, which would occur at a very young age; this increase in TGs has been linked in adults to increased liver fat in non-alcoholic fatty liver disease [104]. In addition, they found decreased total essential TG levels in the plasma of the CD progressors after gluten intake, reversing this trend, but not significantly, after the onset of GFD, and there was an inverse relationship with the tissue transglutaminase IgA titer (tTGA). There was also an increase in cholesterol levels after the start of GFD in the CD progressors. On the other hand, the endogenous TGs plasma levels were decreased in CD progressors independently of gluten intake. PCs were elevated in both CD progressors and controls after the start of gluten intake. A difference in sphingomyelin plasma levels was observed in CD progressors at a later age, after the introduction of GFD.

These findings suggest that a dysregulation in lipid metabolism may be associated with the development of CD, and that it occurs in the first months of life, even before the introduction of gluten to the diet. This could help predict the development of CD in infants at genetic risk, even years before the appearance of specific antibodies or clinical symptoms/signs.

However, a previous study (2016) based on the PreventCD project suggested that the metabolic profile at 4 months (before the introduction of gluten to the diet) did not predict the development of CD, but that metabolic pathways are affected later in life [105]. In this study, which studied serum samples from infants at genetic risk for CD who developed CD compared to those who did not develop the disease at 8 years of age, a trend of decreased phospholipids levels was found in children who subsequently developed CD, although not significantly, with a greater decrease in the subsample of children exclusively breastfed until 4 months of age. They conclude that metabolomic studies should focus on children who have already had gluten introduced to their diet. However, this study focused the analysis on phospholipids and acylcarnitines, and TGs and cholesterol esters were not measured.

Following the lipidomics approach, in a pilot study conducted by ourselves [106], plasma lipid profile was affected in celiac patients, despite GFD treatment. Using an LC-MS/MS platform, there plasma from 17 celiac children under a GFD treatment and 17 healthy controls (siblings) was analyzed. Among the significant molecules, it was found that 64% were increased and 36% decreased in CD patients. Two carboxylic acids and derivatives were increased in CD; other molecules whose levels were affected in patients were four fatty acyls (thromboxanes and leykotrienes involved in inflammatory pathways), five glycerolipids, eleven glycerophospholipids, one organoxigen compound and two sphingolipids, lipid species belonging to steroid metabolism and other molecules involved in bilirubin metabolism. In celiac patients elevated levels of molecules involved in cell signaling pathways (ceramides, diacylglycerides and lysophospholipids) were found. Diacylglycerides play a central role in the control of neuronal communication, phagocytosis and the control of immune responses, and as a second messenger they play an important role in the regulation of mTOR, recently described as a key factor in maintaining a sustained inflammatory response in CD [107].

Aside from the lipid profile, one-carbon metabolism alterations were also found by this group [108] under a targeted plasma metabolomics study. They observed a down-regulation of the trans-sulphuration pathway in CD patients, despite GFD, with decreased cysteine and cystathionine, which, together with normal glutathione and vitamin B6, suggests a specific defect at the level of the enzymes involved in antioxidant defense, oxygen sensing, mitochondrial function, inflammation and second-messenger signaling. This finding, moreover, could be explained by a S-adenosyl-L-homocysteine (SAH) hydrolase mutation that causes typical symptoms of the disease, such as growth retardation, dental anomalies or hypertransaminasemia. In contrast, other pathways involved in one-carbon metabolism appeared to be preserved (choline metabolism, the methionine cycle and the folate cycle), suggesting that adherence to a strict GFD could reverse certain metabolic changes in celiac patients, making them resemble the profile of healthy subjects. This group notes that these metabolomic changes are, however, minor, as only approximately 4% of the total plasma metabolome analyzed was affected [106].

In a more recent study based on a targeted plasma metabolomics analysis, Girdhar et al., 2023 [109] found increased levels of 2-methyl-3-ketovalric acid, taurodeoxycholic acid (TDCA), glucono-D-lactone and isoburyryl-L-carnitine, as well as significantly low oleic acid levels (anti-inflammatory metabolite) in CD progressors (compared to healthy children matched for age, HLA genotype, breastfeeding duration and gluten exposure duration). Other metabolic pathways were also affected in the CD progressors, such as the pentose phosphate pathway, unsaturated fatty acid biosynthesis and glycolipid and linoleic acid metabolism. Notably, TDCA levels were increased to twice the normal values. TDCA, a metabolite derived from the gut microbiota, may play a role in small intestinal inflammation and CD pathogenesis, as its administration to C57BL/6J mice by supplementing their diet caused a distortion in crypt structure and total or partial villous atrophy; increased CD4+ T cells, natural killer cells and Qa-1 and NKG2D expression on T cells (two immunomodulatory proteins); and decreased regulatory T cells in intraepithelial lymphocytes. Therefore, TDCA could be used as an early diagnosis biomarker, and more importantly, targeted therapies to eliminate TDCA-producing bacteria (*Clostridium XIVa* and *Clostridium XI*) early in life could be used as a strategy to decrease the CD development risk. On the other hand, they found that the cytokine plasma profile and other metabolites were altered in CD progressors, even before diagnosis (other recent work (Auricchio et al., 2023 [110]) has also focused on the serum cytokine profile and proinflammatory genes expression in infants at CD risk), and differences were also found in the gut microbiota composition (studied in stool) (other authors have also studied microbiota and metabolome alterations in infants at risk of CD, in stool [111,112]). In the CD progressors, before diagnosis, they found significantly increased levels of three proinflammatory cytokines (IFNA2, IL-1a and IL-17E/(IL25)) and a chemokine (MIP-1b/CCl4).

Another interesting aspect to be addressed is alternative biomarkers that allow the disease to be monitored and can also be used in the evaluation of celiac patients’ relatives. Plasma citrulline was assessed by an LC auto sampler (and in dried blood spots) by Lomash et al., 2021 [113], as a potential biomarker useful in the diagnosis and monitoring of the disease, as well as in the evaluation of celiac patients’ first-degree relatives (FDRs) (predictive value in the distinction of seronegative CD and in the progression of potential to overt CD). This non-essential amino acid is specifically produced by proximal small intestine enterocyte villi, so it has been proposed as a possible marker of residual intestinal function in pathologies such as necrotizing enterocolitis in newborns, enterophaties, small intestine transplantation or small bowel resections [114]. This work found statistically significant differences in the median plasma citrulline levels in celiac children (20.1 μM (IQR, 13.35–29.15)) compared to controls (serology-negative FDRs) (37.33 μM (IQR, 29.8–42.6)). They also found an inverse correlation between plasma citrulline levels and anti-tTG IgA levels throughout the establishment of GFD and, in addition, in the different Marsh grades at diagnosis; so, citrulline could be used as a surrogate biomarker for serology in disease monitoring and in predicting the histopathological damage degree (it was effective in distinguishing grades 3b and above but not in distinguishing 3a or less in celiac patients and healthy asymptomatic FDRs). In addition, in patients with inconclusive serology and biopsy results, the median plasma citrulline reflected mucosal damage (12.26 μM) like in potential celiacs (median plasma citrulline levels: 23.25 μM). A brief summary of the clinical metabolomic studies conducted on the serum and plasma of children with CD is reviewed in Table 2.

### 4.2. Genetic Influence (HLA)

In the above-mentioned work [113], plasma citrulline levels were also correlated with HLA genotype. Significantly low plasma citrulline levels were observed in subjects with the HLA DQ 2.5 genotype with subtypes DQA1*0501 and DQB1*0201. The HLA-DQ genotype has already been reported to influence early intestinal microbial colonization, thus influencing the metabolome [115].

Kirchberg et al. (2016) found that the HLA genotype did not have any influence on the serum metabolic profile in infants who were at risk for celiac disease before introducing gluten to their diet [105].

## 5. Urine Metabolomic Profile

Other studies (Table 3) have also compared the urine metabolomic profile of celiac children with healthy controls, some of them focusing on changes in the volatile organic compounds (VOCs) profile. An example of this is Di Cagno et al., 2011 [116], who, using gas chromatography mass spectrometry/solid-phase microextraction (GC-MS/SPME) analysis, demonstrated that VOCs and free-amino-acid levels are altered in the urine (and stool) of celiac children with more than 2 years of GFD, relating these imbalances to qualitative and quantitative differences in the microbiota of celiac patients compared to healthy people. They found that the CD group had higher dimethyl trisulfide and dimethyl disulfide urine levels. In addition, with some exceptions, they also had higher urine hydrocarbon levels. No differences in urine aldehyde levels were found between the two groups. These findings were confirmed by NMR, which also found that the CD group had higher lysine, arginine, creatine and methylamine mean levels, while carnosine, glucose, glutamine and 3-methyl-2-oxobutanoic acid were the highest in healthy children. This study emphasizes that a GFD does not completely restore the microbiota or, consequently, the metabolome of children with CD, and that there are possible metabolic markers of CD; furthermore, it suggests that the addition of prebiotics and probiotics to the GFD could restore the microbiota–microbiome balance in celiac children.

In relation to this aspect, Drabinska et al., 2019 [117], studied the effect of GFD supplementation with a prebiotic (oligofructose-enriched inulin) on VOC urine concentration in celiac children and adolescents, using GC-MS/SPME analysis. This work is based on the idea that changes in the VOC profile in biological fluids that occur in various gastrointestinal diseases (studied by the recent so-called “volatolomics”) are due in part to alterations in microbiota metabolism, especially its fermentative activity, and not so much to variations in its composition. However, GFD supplementation with this prebiotic had no impact on most VOC urine profiles of celiac patients; only a significant change was observed in benzaldehyde concentrations, which decreased by 36% after 12 weeks of intervention, which may be related to a decrease in *Lactobacillus* counts in the prebiotic-supplemented group, as *Lactobacillus* produces an aminotransferase that converts phenylalanine to benzaldehyde.

Another study, also led by Drabinska [118], aimed to optimize the GC-MS/SPME method for the detection of changes in VOC urine profiles in celiac children compared to healthy children. Based on Variable Importance in the Projection (VIP) scores, several CD biomarkers could be suggested: 1,3-di-tert-butylbenzene (only found in the urine of celiac children) and other VOCs present in higher concentrations in the urine of healthy children (2,3-butanedione, 2-heptanone, dimethyl disulfide and octanal, and, with lower VIP scores, 2-butanone, hexanal and 4-heptanone). Again, these differences could be explained by alterations in the celiac patients’ gut microbiota, as many VOCs are fermentation products of the microbiota. On the other hand, the VOC levels in biological fluids (blood, urine, sweat…) could be increased by the altered intestinal permeability present in many gastrointestinal tract diseases.

## 6. Discussion

Metabolomics is a promising field that offers a comprehensive understanding of cell biology, surpassing other omics sciences in its breadth. However, the current studies in the field indicate that there is still much progress to be made. The limited number of pediatric studies, often pilot studies, that do not consider the impact of a GFD, contribute to these challenges.

Looking ahead, the potential applications of metabolomics are vast. Diagnostic biomarkers for potential celiacs, the normalization of cellular function alongside the proper implementation of a GFD and the identification of unique disease markers hold promise for deeper insights into CD. Many of the studies mentioned in the literature have primarily focused on analyzing metabolite profiles at a single time point, neglecting the potential influence of the GFD on these profiles. Moreover, there is a lack of consensus regarding the definition of cellular normality in patients. Additionally, it remains inconclusive whether a distinct disease footprint exists, although several studies suggest such a possibility. To address these limitations, it is crucial to design comprehensive studies that encompass metabolomic profiles before disease onset, during the disease with consideration of different treatment approaches and in relation to dietary deviations. Such studies hold the potential to identify valuable biomarkers for effective disease management.

Moreover, to advance our understanding, it is crucial to integrate metabolomic studies with other omics sciences, such as transcriptomics, proteomics and the study of the microbiome. Further studies are needed, as well as studies looking at lipid metabolism and other possible biomarkers. This correlation will help unravel the genetic and epigenetic expressions underlying the observed metabolic findings. Furthermore, the role of the GFD, the importance of well-designed research and the correlation with other omics sciences are key factors for comprehending the disease from a metabolomics perspective.

Most of the studies mentioned have fewer than 30 participants. By fostering increased collaboration among these research groups and facilitating the integration of datasets and participant pools, we can pave the way for large-scale metabolomics research endeavors that possess the required statistical power to establish a definitive diagnostic biomarker for celiac disease. By combining resources and expertise, we can overcome the limitations of individual studies and achieve more robust and conclusive results. This collaborative approach holds tremendous potential for advancing our understanding of celiac disease and improving its diagnosis and management.

Unfortunately, while metabolomics presents exciting possibilities, there is still much to be explored. By refining research methodologies and integrating multiple omics sciences, we can unlock a wealth of information and advance our understanding of CD. Future studies, including larger sample sizes and considering various biological tissues beyond plasma, will provide valuable insights.

## 7. Conclusions

CD is a multifactorial entity involving genetic and environmental factors. GFD is the only treatment currently available for CD, although solid biomarkers that allow the adequate monitoring of the disease and treatment are still lacking. On the other hand, it would be interesting to find biomarkers that allow the early diagnosis of the disease. In this sense, metabolomics studies may provide answers to the knowledge gaps that exist in CD and other multifactorial disorders, requiring further research in the pediatric age group.

## Figures and Tables

**Table 1:** Key differences between clinical metabolomics platforms.

	Nuclear Magnetic Resonance (NMR)	Mass Spectrometry (MS)
Sensitivity	Low	High
Dynamic range	Moderate	High
Reproducibility	Very high	Moderate
Detectable metabolites	30–100	300–5000 or more
Metabolite identification	Well categorized	Labor intensive
Targeted analysis	Not optimal	Better than NMR
Sample destructive	Non-destructive	Destructive to sample
Sample preparation	Minimal	More complex than NMR
Tissue extraction	Not required	Required
Sample analysis time	Fast (<10 min)	Longer than NMR (>10 min)
Instrument cost	High	Cheaper than NMR
Sample cost	Low	High

Adapted from different authors [56,57,58,59,60].

**Table 2:** Summary of blood metabolomics findings in the selected studies.

Study Reference	Groups (N)	Age	Sample	Methodology	Key Results
**[102]**	CD progressors (30) vs. HC with similar genetic profiles (20)	0–8 years	Serum	LC-MS, MRM	Altered serum phospholipid profile, even before gluten intake, in CD progressors: Elevated lyso- and PC and PC-O serum levels; Decreased PE 34:1 and PE 36:1 serum levels.
**[103]**	CD progressors (23) vs. HC matched for HLA risk, sex, and age (23)	0–6 years	Plasma	MS	Altered serum lipid profile in CD progressors: Elevated TGs of low carbon number and double-bond count plasma levels. Decreased PC, cholesterol esters, endogenous TGs and total essential TG plasma levels (these latter after gluten intake).
**[105]**	CD progressors (33) vs. HC matched for HLA risk (197)	4 month–8 years	Serum	LC-MS/MS	No significant differences; decreased serum phospholipids levels in CD progressors. No influence of HLA genotype on the serum metabolic profile.
**[106]**	T-CD (17) vs. HC (siblings) (17)	4–17 years	Plasma	LC-MS/MS	Altered plasma lipid profile in T-CD: elevated carboxylic acids and ceramides, diacylglycerides and lysophospholipid plasma levels. Other altered molecules: fatty acyls, glycerolipids, glycerophospholipids, organoxygen compound, sphingolipids, steroid metabolism, molecules involved in bilirubin metabolism.
**[108]**	T-CD (17) vs. HC (siblings) (17)	4–17 years	Plasma	LC-MS/MS	Altered one-carbon metabolism in T-CD: Trans-sulphuration pathway down-regulation (decreased cysteine and cystathionine plasma levels), with glutathione and vitamin B6 normal levels.
**[109]**	CD progressors (7 (plasma samples)) vs. HC matched for age, HLA genotype, breastfeeding duration and gluten exposure duration (9 (plasma samples))	2.5–5 years	Plasma, stool	MS, GC-MS, LC-MS, HR-MS	Altered plasma cytokine profile (and other metabolites) in CD progressors: Elevated IFNA2, IL-1a, IL-17E/(IL25)), MIP-1b/CCl4, 2-Methyl-3-ketovalric acid, TDCA, Glucono-D-lactone and Isoburyryl-L-carnitine; Decreased oleic acid plasma levels.
**[113]**	T-CD (558) vs. HC (FDRs) (1565)	1–18 years (T-CD)	Plasma, DBS	LC-MS, DBS	Decreased plasma citrulline levels in T-CD. Decreased plasma citrulline levels in HLA DQ 2.5-positive patients. Inverse correlation between citrulline levels and anti-tTG IgA levels. Value of citrulline levels as predictors of histopathological damage (Marsh 3b and above).

CD, celiac disease; GFD, gluten-free diet; T-CD, GFD treated celiac disease subjects; HC, healthy controls; CD progressors, children who develop CD; FDRs, first-degree relatives; MS, mass spectrometry; GC-MS, gas chromatography mass spectrometry; LC-MS, liquid chromatography coupled with mass spectrometry; HR-MS, high resolution mass spectrometry; MRM, multiple-reaction monitoring; DBS, dried blood spot; PC, phosphatidylcholines; PC-O, alkylacylphosphatidylcholines; PE, phosphatidylethanolamines; TGs, triacylgycerols; TDCA, taurodeoxycholic acid; HLA, human leukocyte antigen; anti-tTG IgA, anti-tissue transglutaminase IgA.

**Table 3:** Summary of urine metabolomics findings in the selected studies.

Study Reference	Groups (N)	Age	Sample	Methodology	Key Results
**[116]**	T-CD (19) vs. HC (15)	6–12 years	Stool, urine	GC-MS/SPME, H-NMR	Altered VOCs and free-amino-acid levels: - T-CD: elevated dimethyl trisulfide and dimethyl disulfide urine levels and most hydrocarbon levels. Elevated lysine, arginine, creatine and methylamine mean levels: - HC: elevated carnosine, glucose, glutamine and 3-methyl-2-oxobutanoic acid levels.
**[117]**	T-CD Synergy 1 (11) vs. T-CD placebo (12)	4–18 years	Urine	GC-MS/SPME	No significant changes in VOC urine profiles, except for benzaldehyde concentrations (36% decrease after 12 weeks of intervention).
**[118]**	T-CD (9) vs. HC (9)	4–14 years	Urine	GC-MS/SPME	Altered VOC levels: - Only in T-CD: 1,3-di-tert-butylbenzene in urine. - HC: elevated 2,3-butanedione, 2-heptanone, dimethyl disulfide and octanal levels (and 2-butanone, hexanal and 4-heptanone).

CD, celiac disease; GFD, gluten-free diet; T-CD, GFD treated celiac disease subjects; HC, healthy controls; GC-MS/SPME, gas chromatography mass spectrometry/solid-phase microextraction; H-NMR, hydrogen-1 nuclear magnetic resonance; VOCs, volatile organic compounds.

## Data Availability

Not applicable.

## References

[1] Martín-Masot R., Diaz-Castro J., Moreno-Fernandez J., Navas-López V.M., Nestares T. (2020). The Role of Early Programming and Early Nutrition on the Development and Progression of Celiac Disease: A Review. Nutrients.

[2] Ludvigsson J.F., Bai J.C., Biagi F., Card T.R., Ciacci C., Ciclitira P.J., Green P.H.R., Hadjivassiliou M., Holdoway A., Van Heel D.A. (2014). Diagnosis and Management of Adult Coeliac Disease: Guidelines from the British Society of Gastroenterology. Gut.

[3] Szajewska H., Shamir R., Mearin L., Ribes-Koninckx C., Catassi C., Domellof M., Fewtrell M.S., Husby S., Papadopoulou A., Vandenplas Y. (2016). Gluten Introduction and the Risk of Coeliac Disease: A Position Paper by the European Society for Pediatric Gastroenterology, Hepatology, and Nutrition. J. Pediatr. Gastroenterol. Nutr..

[4] Lebwohl B., Sanders D.S., Green P.H.R. (2018). Coeliac Disease. Lancet.

[5] Caio G., Volta U., Sapone A., Leffler D.A., De Giorgio R., Catassi C., Fasano A. (2019). Celiac Disease: A Comprehensive Current Review. BMC Med..

[6] Leonard M.M., Karathia H., Pujolassos M., Troisi J., Valitutti F., Subramanian P., Camhi S., Kenyon V., Colucci A., Serena G. (2020). Multi-Omics Analysis Reveals the Influence of Genetic and Environmental Risk Factors on Developing Gut Microbiota in Infants at Risk of Celiac Disease. Microbiome.

[7] King J.A., Jeong J., Underwood F.E., Quan J., Panaccione N., Windsor J.W., Coward S., Debruyn J., Ronksley P.E., Shaheen A.A. (2020). Incidence of Celiac Disease Is Increasing over Time: A Systematic Review and Meta-Analysis. Am. J. Gastroenterol..

[8] Abrams J.A., Diamond B., Rotterdam H., Green P.H.R. (2004). Seronegative Celiac Disease: Increased Prevalence with Lesser Degrees of Villous Atrophy. Dig. Dis. Sci..

[9] Charlesworth R.P.G. (2020). Diagnosing Coeliac Disease: Out with the Old and in with the New?. World J. Gastroenterol..

[10] Simpson S.M., Ciaccio E.J., Case S., Jaffe N., Mahadov S., Lebwohl B., Green P.H. (2013). Celiac Disease in Patients with Type 1 Diabetes: Screening and Diagnostic Practices. Diabetes Educ..

[11] Volta U., Rostami K., Tovoli F., Caio G., Masi C., Ruggeri E., Cacciari G., Bon I., De Giorgio R. (2013). Fulminant Type 1 Autoimmune Hepatitis in a Recently Diagnosed Celiac Disease Patient. Arch. Iran Med..

[12] Martín-Masot R., Herrador-López M., Navas-López V.M., Carmona F.D., Nestares T., Bossini-Castillo L. (2023). Celiac Disease Is a Risk Factor for Mature T and NK Cell Lymphoma: A Mendelian Randomization Study. Int. J. Mol. Sci..

[13] Diaz-Castro J., Muriel-Neyra C., Martin-Masot R., Moreno-Fernandez J., Maldonado J., Nestares T. (2019). Oxidative Stress, DNA Stability and Evoked Inflammatory Signaling in Young Celiac Patients Consuming a Gluten-Free Diet. Eur. J. Nutr..

[14] Nestares T., Martín-Masot R., Flor-Alemany M., Bonavita A., Maldonado J., Aparicio V.A. (2021). Influence of Ultra-Processed Foods Consumption on Redox Status and Inflammatory Signaling in Young Celiac Patients. Nutrients.

[15] Nestares T., Martín-Masot R., Labella A., Aparicio V.A., Flor-Alemany M., López-Frías M., Maldonado J. (2020). Is a Gluten-Free Diet Enough to Maintain Correct Micronutrients Status in Young Patients with Celiac Disease?. Nutrients.

[16] Silano M., Volta U., Mecchia A.M., Dessì M., Di Benedetto R., De Vincenzi M. (2007). Delayed Diagnosis of Coeliac Disease Increases Cancer Risk. BMC Gastroenterol..

[17] Viljamaa M., Kaukinen K., Pukkala E., Hervonen K., Reunala T., Collin P. (2006). Malignancies and Mortality in Patients with Coeliac Disease and Dermatitis Herpetiformis: 30-Year Population-Based Study. Dig. Liver Dis..

[18] Mearin M.L., Catassi C., Brousse N., Brand R., Collin P., Fabiani E., Schweizer J.J., Abuzakouk M., Szajewska H., Hallert C. (2006). European Multi-Centre Study on Coeliac Disease and Non-Hodgkin Lymphoma. Eur. J. Gastroenterol. Hepatol..

[19] Ilus T., Kaukinen K., Virta L.J., Pukkala E., Collin P. (2014). Incidence of Malignancies in Diagnosed Celiac Patients: A Population-Based Estimate. Am. J. Gastroenterol..

[20] Kell D.B., Goodacre R. (2014). Metabolomics and Systems Pharmacology: Why and How to Model the Human Metabolic Network for Drug Discovery. Drug Discov. Today.

[21] Allen J., Davey H.M., Broadhurst D., Heald J.K., Rowland J.J., Oliver S.G., Kell D.B. (2003). High-Throughput Classification of Yeast Mutants for Functional Genomics Using Metabolic Footprinting. Nat. Biotechnol..

[22] Sellitto M., Bai G., Serena G., Fricke W.F., Sturgeon C., Gajer P., White J.R., Koenig S.S.K., Sakamoto J., Boothe D. (2012). Proof of Concept of Microbiome-Metabolome Analysis and Delayed Gluten Exposure on Celiac Disease Autoimmunity in Genetically at-Risk Infants. PLoS ONE.

[23] Nicholson J.K., Lindon J.C., Holmes E. (1999). “Metabonomics”: Understanding the Metabolic Responses of Living Systems to Pathophysiological Stimuli via Multivariate Statistical Analysis of Biological NMR Spectroscopic Data. Xenobiotica.

[24] Evans A.M., DeHaven C.D., Barrett T., Mitchell M., Milgram E. (2009). Integrated, Nontargeted Ultrahigh Performance Liquid Chromatography/Electrospray Ionization Tandem Mass Spectrometry Platform for the Identification and Relative Quantification of the Small-Molecule Complement of Biological Systems. Anal. Chem..

[25] Dunn W.B. (2008). Current Trends and Future Requirements for the Mass Spectrometric Investigation of Microbial, Mammalian and Plant Metabolomes. Phys. Biol..

[26] Kan M., Himes B.E. (2021). Insights into Glucocorticoid Responses Derived from Omics Studies. Pharmacol. Ther..

[27] Bordag N., Klie S., Jürchott K., Vierheller J., Schiewe H., Albrecht V., Tonn J.-C., Schwartz C., Schichor C., Selbig J. (2015). Glucocorticoid (Dexamethasone)-Induced Metabolome Changes in Healthy Males Suggest Prediction of Response and Side Effects. Sci. Rep..

[28] Wishart D.S. (2016). Emerging Applications of Metabolomics in Drug Discovery and Precision Medicine. Nat. Rev. Drug Discov..

[29] Johnson C.H., Ivanisevic J., Siuzdak G. (2016). Metabolomics: Beyond Biomarkers and towards Mechanisms. Nat. Rev. Mol. Cell Biol..

[30] Petrick L., Edmands W., Schiffman C., Grigoryan H., Perttula K., Yano Y., Dudoit S., Whitehead T., Metayer C., Rappaport S. (2017). An Untargeted Metabolomics Method for Archived Newborn Dried Blood Spots in Epidemiologic Studies. Metabolomics.

[31] Niedzwiecki M.M., Walker D.I., Vermeulen R., Chadeau-Hyam M., Jones D.P., Miller G.W. (2019). The Exposome: Molecules to Populations. Annu. Rev. Pharmacol. Toxicol..

[32] Johnson J.M., Yu T., Strobel F.H., Jones D.P. (2010). A Practical Approach to Detect Unique Metabolic Patterns for Personalized Medicine. Analyst.

[33] Cabré R., Jové M., Naudí A., Ayala V., Piñol-Ripoll G., Gil-Villar M.P., Dominguez-Gonzalez M., Obis È., Berdun R., Mota-Martorell N. (2016). Specific Metabolomics Adaptations Define a Differential Regional Vulnerability in the Adult Human Cerebral Cortex. Front. Mol. Neurosci..

[34] Goodpaster B.H., Sparks L.M. (2017). Metabolic Flexibility in Health and Disease. Cell Metab..

[35] Moreau R., Clària J., Aguilar F., Fenaille F., Lozano J.J., Junot C., Colsch B., Caraceni P., Trebicka J., Pavesi M. (2020). Blood Metabolomics Uncovers Inflammation-Associated Mitochondrial Dysfunction as a Potential Mechanism Underlying ACLF. J. Hepatol..

[36] Pang H., Jia W., Hu Z. (2019). Emerging Applications of Metabolomics in Clinical Pharmacology. Clin. Pharmacol. Ther..

[37] Zhang A., Sun H., Wang X. (2013). Power of Metabolomics in Biomarker Discovery and Mining Mechanisms of Obesity. Obes. Rev..

[38] Thomson T.M., Balcells C., Cascante M. (2019). Metabolic Plasticity and Epithelial-Mesenchymal Transition. J. Clin. Med..

[39] Saner C., Harcourt B.E., Pandey A., Ellul S., McCallum Z., Kao K.-T., Twindyakirana C., Pons A., Alexander E.J., Saffery R. (2019). Sex and Puberty-Related Differences in Metabolomic Profiles Associated with Adiposity Measures in Youth with Obesity. Metabolomics.

[40] Handelman S.K., Romero R., Tarca A.L., Pacora P., Ingram B., Maymon E., Chaiworapongsa T., Hassan S.S., Erez O. (2019). The Plasma Metabolome of Women in Early Pregnancy Differs from That of Non-Pregnant Women. PLoS ONE.

[41] Christian P., Smith E.R., Lee S.E., Vargas A.J., Bremer A.A., Raiten D.J. (2021). The Need to Study Human Milk as a Biological System. Am. J. Clin. Nutr..

[42] Mota-Martorell N., Jové M., Borrás C., Berdún R., Obis È., Sol J., Cabré R., Pradas I., Galo-Licona J.D., Puig J. (2021). Methionine Transsulfuration Pathway Is Upregulated in Long-Lived Humans. Free Radic. Biol. Med..

[43] Sol J., Obis È., Mota-Martorell N., Pradas I., Galo-Licona J.D., Martin-Garí M., Fernández-Bernal A., Ortega-Bravo M., Mayneris-Perxachs J., Borrás C. (2023). Plasma Acylcarnitines and Gut-derived Aromatic Amino Acids as Sex-specific Hub Metabolites of the Human Aging Metabolome. Aging Cell.

[44] Sas K.M., Karnovsky A., Michailidis G., Pennathur S. (2015). Metabolomics and Diabetes: Analytical and Computational Approaches. Diabetes.

[45] Wang-Sattler R., Yu Z., Herder C., Messias A.C., Floegel A., He Y., Heim K., Campillos M., Holzapfel C., Thorand B. (2012). Novel Biomarkers for Pre-diabetes Identified by Metabolomics. Mol. Syst. Biol..

[46] Pallares-Méndez R., Aguilar-Salinas C.A., Cruz-Bautista I., del Bosque-Plata L. (2016). Metabolomics in Diabetes, a Review. Ann. Med..

[47] Danzi F., Pacchiana R., Mafficini A., Scupoli M.T., Scarpa A., Donadelli M., Fiore A. (2023). To Metabolomics and beyond: A Technological Portfolio to Investigate Cancer Metabolism. Signal Transduct. Target Ther..

[48] Gatius S., Jove M., Megino-Luque C., Albertí-Valls M., Yeramian A., Bonifaci N., Piñol M., Santacana M., Pradas I., Llobet-Navas D. (2022). Metabolomic Analysis Points to Bioactive Lipid Species and Acireductone Dioxygenase 1 (ADI1) as Potential Therapeutic Targets in Poor Prognosis Endometrial Cancer. Cancers.

[49] Shao Y., Le W. (2019). Recent Advances and Perspectives of Metabolomics-Based Investigations in Parkinson’s Disease. Mol. Neurodegener..

[50] Pamplona R., Obis E., Sol J., Andres-Benito P., Martí n-Gari M., Mota-Martorell N., Daniel Galo-Licona J., Piñol-Ripoll G., Portero-Otin M., Ferrer I. (2023). Lipidomic Alterations in the Cerebral Cortex and White Matter in Sporadic Alzheimer’s Disease. bioRxiv.

[51] Centanni M., Moes D.J.A.R., Trocóniz I.F., Ciccolini J., van Hasselt J.G.C. (2019). Clinical Pharmacokinetics and Pharmacodynamics of Immune Checkpoint Inhibitors. Clin. Pharmacokinet..

[52] Alarcon-Barrera J.C., Kostidis S., Ondo-Mendez A., Giera M. (2022). Recent Advances in Metabolomics Analysis for Early Drug Development. Drug Discov. Today.

[53] De Castro F., Benedetti M., Del Coco L., Fanizzi F.P. (2019). NMR-Based Metabolomics in Metal-Based Drug Research. Molecules.

[54] Lindon J.C., Holmes E., Nicholson J.K. (2007). Metabonomics in Pharmaceutical R & D. FEBS J..

[55] Uppal K., Walker D.I., Liu K., Li S., Go Y.-M., Jones D.P. (2016). Computational Metabolomics: A Framework for the Million Metabolome. Chem. Res. Toxicol..

[56] Yoon D., Lee M., Kim S., Kim S. (2013). Applications of NMR Spectroscopy Based Metabolomics: A Review. J. Korean Magn. Reson. Soc..

[57] Kim J.U., Shariff M.I.F., Crossey M.M.E., Gomez-Romero M., Holmes E., Cox I.J., Fye H.K.S., Njie R., Taylor-Robinson S.D. (2016). Hepatocellular Carcinoma: Review of Disease and Tumor Biomarkers. World J. Hepatol..

[58] Emwas A.-H.M. (2015). The Strengths and Weaknesses of NMR Spectroscopy and Mass Spectrometry with Particular Focus on Metabolomics Research. Metabonomics: Methods and Protocols.

[59] Adamski J. (2020). Introduction to Metabolomics. Metabolomics for Biomedical Research.

[60] Bhinderwala F., Wase N., DiRusso C., Powers R. (2018). Combining Mass Spectrometry and NMR Improves Metabolite Detection and Annotation. J. Proteome Res..

[61] Emwas A.-H., Roy R., McKay R.T., Tenori L., Saccenti E., Gowda G.A.N., Raftery D., Alahmari F., Jaremko L., Jaremko M. (2019). NMR Spectroscopy for Metabolomics Research. Metabolites.

[62] Keun H.C., Athersuch T.J. (2011). Nuclear Magnetic Resonance (NMR)-Based Metabolomics. Metabolic Profiling: Methods and Protocols.

[63] Letertre M.P.M., Dervilly G., Giraudeau P. (2021). Combined Nuclear Magnetic Resonance Spectroscopy and Mass Spectrometry Approaches for Metabolomics. Anal. Chem..

[64] Psychogios N., Hau D.D., Peng J., Guo A.C., Mandal R., Bouatra S., Sinelnikov I., Krishnamurthy R., Eisner R., Gautam B. (2011). The Human Serum Metabolome. PLoS ONE.

[65] Junot C., Fenaille F., Colsch B., Bécher F. (2014). High Resolution Mass Spectrometry Based Techniques at the Crossroads of Metabolic Pathways. Mass Spectrom. Rev..

[66] Yanes O., Tautenhahn R., Patti G.J., Siuzdak G. (2011). Expanding Coverage of the Metabolome for Global Metabolite Profiling. Anal. Chem..

[67] Piraud M., Vianey-Saban C., Petritis K., Elfakir C., Steghens J., Morla A., Bouchu D. (2003). ESI-MS/MS Analysis of Underivatised Amino Acids: A New Tool for the Diagnosis of Inherited Disorders of Amino Acid Metabolism. Fragmentation Study of 79 Molecules of Biological Interest in Positive and Negative Ionisation Mode. Rapid Commun. Mass Spectrom..

[68] Bruins A.P. (2005). Mass spectrometry—Atmospheric Pressure Ionization Techniques. Encyclopedia of Analytical Science.

[69] Schuhmacher J., Zimmer D., Tesche F., Pickard V. (2003). Matrix Effects during Analysis of Plasma Samples by Electrospray and Atmospheric Pressure Chemical Ionization Mass Spectrometry: Practical Approaches to Their Elimination. Rapid Commun. Mass Spectrom..

[70] Karas M., Krüger R. (2003). Ion Formation in MALDI: The Cluster Ionization Mechanism. Chem. Rev..

[71] Theodoridis G., Gika H.G., Wilson I.D. (2011). Mass Spectrometry-Based Holistic Analytical Approaches for Metabolite Profiling in Systems Biology Studies. Mass Spectrom. Rev..

[72] Want E.J., Masson P., Michopoulos F., Wilson I.D., Theodoridis G., Plumb R.S., Shockcor J., Loftus N., Holmes E., Nicholson J.K. (2013). Global Metabolic Profiling of Animal and Human Tissues via UPLC-MS. Nat. Protoc..

[73] Špánik I., Machyňáková A. (2018). Recent Applications of Gas Chromatography with High-Resolution Mass Spectrometry. J. Sep. Sci..

[74] Zeki Ö.C., Eylem C.C., Reçber T., Kır S., Nemutlu E. (2020). Integration of GC–MS and LC–MS for Untargeted Metabolomics Profiling. J. Pharm. Biomed. Anal..

[75] Marshall D.D., Powers R. (2017). Beyond the Paradigm: Combining Mass Spectrometry and Nuclear Magnetic Resonance for Metabolomics. Prog. Nucl. Magn. Reason. Spectrosc..

[76] Martias C., Baroukh N., Mavel S., Blasco H., Lefèvre A., Roch L., Montigny F., Gatien J., Schibler L., Dufour-Rainfray D. (2021). Optimization of Sample Preparation for Metabolomics Exploration of Urine, Feces, Blood and Saliva in Humans Using Combined NMR and UHPLC-HRMS Platforms. Molecules.

[77] Vuckovic D. (2013). Sample Preparation in Global Metabolomics of Biological Fluids and Tissues. Proteomic and Metabolomic Approaches to Biomarker Discovery.

[78] Lu W., Bennett B.D., Rabinowitz J.D. (2008). Analytical Strategies for LC–MS-Based Targeted Metabolomics. J. Chromatogr. B.

[79] Patti G.J., Tautenhahn R., Siuzdak G. (2012). Meta-Analysis of Untargeted Metabolomic Data from Multiple Profiling Experiments. Nat. Protoc..

[80] Jacob M., Malkawi A., Albast N., Al Bougha S., Lopata A., Dasouki M., Abdel Rahman A.M. (2018). A Targeted Metabolomics Approach for Clinical Diagnosis of Inborn Errors of Metabolism. Anal. Chim. Acta.

[81] Puigarnau S., Fernàndez A., Obis E., Jové M., Castañer M., Pamplona R., Portero-Otin M., Camerino O. (2022). Metabolomics Reveals That Fittest Trail Runners Show a Better Adaptation of Bioenergetic Pathways. J. Sci. Med. Sport.

[82] Dunn W.B., Broadhurst D., Begley P., Zelena E., Francis-McIntyre S., Anderson N., Brown M., Knowles J.D., Halsall A., Haselden J.N. (2011). Procedures for Large-Scale Metabolic Profiling of Serum and Plasma Using Gas Chromatography and Liquid Chromatography Coupled to Mass Spectrometry. Nat. Protoc..

[83] Molnos S., Wahl S., Haid M., Eekhoff E.M.W., Pool R., Floegel A., Deelen J., Much D., Prehn C., Breier M. (2018). Metabolite Ratios as Potential Biomarkers for Type 2 Diabetes: A DIRECT Study. Diabetologia.

[84] Newgard C.B. (2017). Metabolomics and Metabolic Diseases: Where Do We Stand?. Cell Metab..

[85] Phapale P. (2021). Pharmaco-metabolomics Opportunities in Drug Development and Clinical Research. Anal. Sci. Adv..

[86] Zhang M., Yang H. (2022). Perspectives from Metabolomics in the Early Diagnosis and Prognosis of Gestational Diabetes Mellitus. Front. Endocrinol..

[87] Mickiewicz B., Vogel H.J., Wong H.R., Winston B.W. (2013). Metabolomics as a Novel Approach for Early Diagnosis of Pediatric Septic Shock and Its Mortality. Am. J. Respir Crit. Care Med..

[88] Kaddurah-Daouk R., Krishnan K.R.R. (2009). Metabolomics: A Global Biochemical Approach to the Study of Central Nervous System Diseases. Neuropsychopharmacology.

[89] Mock A., Zschäbitz S., Kirsten R., Scheffler M., Wolf B., Herold-Mende C., Kramer R., Busch E., Jenzer M., Jäger D. (2019). Serum Very Long-Chain Fatty Acid-Containing Lipids Predict Response to Immune Checkpoint Inhibitors in Urological Cancers. Cancer Immunol. Immunother..

[90] Paglia G., Williams J.P., Menikarachchi L., Thompson J.W., Tyldesley-Worster R., Halldórsson S., Rolfsson O., Moseley A., Grant D., Langridge J. (2014). Ion Mobility Derived Collision Cross Sections to Support Metabolomics Applications. Anal. Chem..

[91] Paglia G., Kliman M., Claude E., Geromanos S., Astarita G. (2015). Applications of Ion-Mobility Mass Spectrometry for Lipid Analysis. Anal. Bioanal. Chem..

[92] Paglia G., Smith A.J., Astarita G. (2022). Ion Mobility Mass Spectrometry in the Omics Era: Challenges and Opportunities for Metabolomics and Lipidomics. Mass Spectrom. Rev..

[93] Heuillet M., Bellvert F., Cahoreau E., Letisse F., Millard P., Portais J.-C. (2018). Methodology for the Validation of Isotopic Analyses by Mass Spectrometry in Stable-Isotope Labeling Experiments. Anal. Chem..

[94] Winter G., Krömer J.O. (2013). Fluxomics—Connecting ‘omics Analysis and Phenotypes. Environ. Microbiol..

[95] Emwas A.-H., Szczepski K., Al-Younis I., Lachowicz J.I., Jaremko M. (2022). Fluxomics—New Metabolomics Approaches to Monitor Metabolic Pathways. Front. Pharmacol..

[96] Jang C., Chen L., Rabinowitz J.D. (2018). Metabolomics and Isotope Tracing. Cell.

[97] Eggers L.F., Schwudke D. (2018). Shotgun Lipidomics Approach for Clinical Samples. Methods Mol. Biol..

[98] Chekmeneva E., dos Santos Correia G., Chan Q., Wijeyesekera A., Tin A., Young J.H., Elliott P., Nicholson J.K., Holmes E. (2017). Optimization and Application of Direct Infusion Nanoelectrospray HRMS Method for Large-Scale Urinary Metabolic Phenotyping in Molecular Epidemiology. J. Proteom. Res..

[99] Taylor M.J., Lukowski J.K., Anderton C.R. (2021). Spatially Resolved Mass Spectrometry at the Single Cell: Recent Innovations in Proteomics and Metabolomics. J. Am. Soc. Mass Spectrom..

[100] Miura D., Fujimura Y., Wariishi H. (2012). In Situ Metabolomic Mass Spectrometry Imaging: Recent Advances and Difficulties. J. Proteom..

[101] Abu Sammour D., Cairns J.L., Boskamp T., Marsching C., Kessler T., Ramallo Guevara C., Panitz V., Sadik A., Cordes J., Schmidt S. (2023). Spatial Probabilistic Mapping of Metabolite Ensembles in Mass Spectrometry Imaging. Nat. Commun..

[102] Auricchio R., Galatola M., Cielo D., Amoresano A., Caterino M., De Vita E., Illiano A., Troncone R., Greco L., Ruoppolo M. (2019). A Phospholipid Profile at 4 Months Predicts the Onset of Celiac Disease in At-Risk Infants. Sci. Rep..

[103] Sen P., Carlsson C., Virtanen S.M., Simell S., Hyöty H., Ilonen J., Toppari J., Veijola R., Hyötyläinen T., Knip M. (2019). Persistent Alterations in Plasma Lipid Profiles Before Introduction of Gluten in the Diet Associated with Progression to Celiac Disease. Clin. Transl. Gastroenterol..

[104] Orešič M., Hyötyläinen T., Kotronen A., Gopalacharyulu P., Nygren H., Arola J., Castillo S., Mattila I., Hakkarainen A., Borra R.J.H. (2013). Prediction of Non-Alcoholic Fatty-Liver Disease and Liver Fat Content by Serum Molecular Lipids. Diabetologia.

[105] Kirchberg F.F., Werkstetter K.J., Uhl O., Auricchio R., Castillejo G., Korponay-Szabo I.R., Polanco I., Ribes-Koninckx C., Vriezinga S.L., Koletzko B. (2016). Investigating the Early Metabolic Fingerprint of Celiac Disease—A Prospective Approach. J. Autoimmun..

[106] Martín-Masot R., Galo-Licona J.D., Mota-Martorell N., Sol J., Jové M., Maldonado J., Pamplona R., Nestares T. (2021). Up-Regulation of Specific Bioactive Lipids in Celiac Disease. Nutrients.

[107] Sedda S., Dinallo V., Marafini I., Franzè E., Paoluzi O.A., Izzo R., Giuffrida P., Di Sabatino A., Corazza G.R., Monteleone G. (2020). MTOR Sustains Inflammatory Response in Celiac Disease. Sci. Rep..

[108] Martín-Masot R., Mota-Martorell N., Jové M., Maldonado J., Pamplona R., Nestares T. (2020). Alterations in One-Carbon Metabolism in Celiac Disease. Nutrients.

[109] Girdhar K., Dogru Y.D., Huang Q., Yang Y., Tolstikov V., Raisingani A., Chrudinova M., Oh J., Kelley K., Ludvigsson J.F. (2023). Dynamics of the Gut Microbiome, IgA Response, and Plasma Metabolome in the Development of Pediatric Celiac Disease. Microbiome.

[110] Auricchio R., Galatola M., Cielo D., Rotondo R., Carbone F., Mandile R., Carpinelli M., Vitale S., Matarese G., Gianfrani C. (2023). Antibody Profile, Gene Expression and Serum Cytokines in At-Risk Infants before the Onset of Celiac Disease. Int. J. Mol. Sci..

[111] Fasano A., Leonard M.M., Kenyon V., Valitutti F., Pennacchio-Harrington R., Piemontese P., Francavilla R., Norsa L., Passaro T., Crocco M. (2023). Cohort Profile: Celiac Disease Genomic, Environmental, Microbiome and Metabolome Study; a Prospective Longitudinal Birth Cohort Study of Children at-Risk for Celiac Disease. PLoS ONE.

[112] Leonard M.M., Valitutti F., Karathia H., Pujolassos M., Kenyon V., Fanelli B., Troisi J., Subramanian P., Camhi S., Colucci A. (2021). Microbiome Signatures of Progression toward Celiac Disease Onset in At-Risk Children in a Longitudinal Prospective Cohort Study. Proc. Natl. Acad. Sci. USA.

[113] Lomash A., Prasad A., Singh R., Kumar S., Gupta R., Dholakia D., Kumar P., Batra V.V., Puri A.S., Kapoor S. (2021). Evaluation of the Utility of Amino Acid Citrulline as a Surrogate Metabolomic Biomarker for the Diagnosis of Celiac Disease. Nutr. Metab. Insights.

[114] Crenn P., Messing B., Cynober L. (2008). Citrulline as a Biomarker of Intestinal Failure Due to Enterocyte Mass Reduction. Clin. Nutr..

[115] Olivares M., Albrecht S., De Palma G., Ferrer M.D., Castillejo G., Schols H.A., Sanz Y. (2015). Human Milk Composition Differs in Healthy Mothers and Mothers with Celiac Disease. Eur. J. Nutr..

[116] Di Cagno R., De Angelis M., De Pasquale I., Ndagijimana M., Vernocchi P., Ricciuti P., Gagliardi F., Laghi L., Crecchio C., Guerzoni M. (2011). Duodenal and Faecal Microbiota of Celiac Children: Molecular, Phenotype and Metabolome Characterization. BMC Microbiol..

[117] Drabinska N., Jarocka-Cyrta E., Ratcliffe N.M., Krupa-Kozak U. (2019). The Profile of Urinary Headspace Volatile Organic Compounds After 12-Week Intake of Oligofructose-Enriched Inulin by Children and Adolescents with Celiac Disease on a Gluten-Free Diet: Results of a Pilot, Randomized, Placebo-Controlled Clinical Trial. Molecules.

[118] Drabińska N., Azeem H.A., Krupa-Kozak U. (2018). A Targeted Metabolomic Protocol for Quantitative Analysis of Volatile Organic Compounds in Urine of Children with Celiac Disease. RSC Adv..

